# A Prognostic Model Based on mRNA Expression Analysis of Esophageal Squamous Cell Carcinoma

**DOI:** 10.3389/fbioe.2022.823619

**Published:** 2022-03-01

**Authors:** Ke Liu, Ye-Lin Jiao, Liu-Qing Shen, Pan Chen, Ying Zhao, Meng-Xiang Li, Bian-Li Gu, Zi-Jun Lan, Hao-Jie Ruan, Qi-Wei Liu, Feng-Bo Xu, Xiang Yuan, Yi-Jun Qi, She-Gan Gao

**Affiliations:** ^1^ School of Information Engineering, Henan University of Science and Technology, Luoyang, China; ^2^ Henan Key Laboratory of Microbiome and Esophageal Cancer Prevention and Treatment, Henan Key Laboratory of Cancer Epigenetics, Cancer Hospital, The First Affiliated Hospital (College of Clinical Medicine) of Henan University of Science and Technology, Luoyang, China; ^3^ Department of Pathology, Luo Yang First Peoples’s Hospital, Luoyang, China

**Keywords:** esophageal squamous cell carcinoma, marker gene, nomogram, prognosis, dimensionality reduction (DR)

## Abstract

**Background:** The aim of this study was to identify prognostic markers for esophageal squamous cell carcinoma (ESCC) and build an effective prognostic nomogram for ESCC.

**Methods:** A total of 365 patients with ESCC from three medical centers were divided into four cohorts. In the discovery phase of the study, we analyzed transcriptional data from 179 cancer tissue samples and identified nine marker genes using edgeR and rbsurv packages. In the training phase, penalized Cox regression was used to select the best marker genes and clinical characteristics in the 179 samples. In the verification phase, these marker genes and clinical characteristics were verified by internal validation cohort (n = 58) and two external cohorts (*n* = 81, *n* = 105).

**Results:** We constructed and verified a nomogram model based on multiple clinicopathologic characteristics and gene expression of a patient cohort undergoing esophagectomy and adjuvant radiochemotherapy. The predictive accuracy for 4-year overall survival (OS) indicated by the C-index was 0.75 (95% CI, 0.72–0.78), which was statistically significantly higher than that of the American Joint Committee on Cancer (AJCC) seventh edition (0.65). Furthermore, we found two marker genes (TM9SF1, PDZK1IP) directly related to the OS of esophageal cancer.

**Conclusion:** The nomogram presented in this study can accurately and impersonally predict the prognosis of ESCC patients after partial resection of the esophagus. More research is required to determine whether it can be applied to other patient populations. Moreover, we found two marker genes directly related to the prognosis of ESCC, which will provide a basis for future research.

## Introduction

Esophageal cancer (EC) is a very common digestive tract tumor with the sixth highest mortality rate in the world, and there are about 150,000 deaths from EC in China every year. (Rubenstein and Shaheen, 2015; Liang et al., 2017) The histological types of EC mainly include esophageal squamous cell carcinoma (ESCC) and esophageal adenocarcinoma (EAC). In 2018, more than 570,000 people worldwide were diagnosed with EC, and more than 500,000 people died of EC in the same year. (Enzinger and Mayer, 2003) Most of the new cases and deaths in the world occur in less developed areas. (Gao et al., 2018) Histologically, approximately 90% of EC cases in China are ESCC. (International Agency for Research on Cnacer, 2012) ESCC is characterized by high aggressiveness and poor prognosis. (Qi et al., 2012) Despite various comprehensive treatments, including surgery, radiotherapy, and chemotherapy, the 5-year survival rate of patients is still less than 22%. (Zhao et al., 2012) The significant geographical variation in incidence means that environmental and genetic factors could play major roles in the development of EC. Smoking and drinking are as known as risk factors for EC, whereas high consumption of vegetables and fruits is likely to prevent EC. (Ando, 2015; Owen et al., 2018; Wu et al., 2018)

At present, the tumor-node-metastasis (TNM) staging system ignores the important clinical factors of tumor prognosis, and the great difference in clinical course leads to the inaccuracy of TNM staging, so it is necessary to establish a new ESCC prognosis grading system. (Cao et al., 2016; Duan et al., 2016) A nomogram can successfully quantify risk prediction by incorporating and illustrating important factors for tumor prognosis. (Wierda et al., 2007; Zhang et al., 2018; Sun et al., 2019) Compared with the TNM staging system, a nomogram can not only predict the survival of all types of cancer patients more accurately but also quantify the outcome of survival prediction by using clinical factors and other factors affecting the prognosis of cancer. Thus, the nomogram is a new prognostic criterion that produces a quantified risk probability of clinical survival by creating a linear graph of the prediction model instead of the traditional method. (Mariani et al., 2005; Sternberg, 2006; Wang et al., 2006) We hypothesized that combining multiple clinicopathologic characteristics and signature gene expression levels can improve the prediction result of ESCC, but there remains a paucity of reliable genetic markers. TGF-β1 is an efficient prognostic biomarker for ESCC patients. HER-2 can be used as a potential molecular marker for ESCC molecular typing. But, HER-2 is not an efficient prognostic biomarker and potential therapeutic target for Iranian ESCC patients. (Heidarpour et al., 2020) Using the partial likelihood of the Cox model, we recently excavated a gene set that is closely related to the overall survival (OS) of ESCC patients.

To the best of the authors’ knowledge, this paper presents the first ESCC nomogram model based on multiple clinicopathologic characteristics and gene expression of a patient cohort undergoing esophagectomy and adjuvant radiochemotherapy. Furthermore, we used an independent cohort from The Cancer Genome Atlas (TCGA) database for external validation. Another independent cohort of 105 ESCC patients was employed to verify the effectiveness of the gene that we found. This study also compared the nomogram with the TNM staging system, proving that the model is more effective in survival prediction.

## Materials and Methods

### Patient Selection

We downloaded transcriptome sequencing data of 179 ESCC samples from the GEO database (GSE53625) (Li et al., 2014). This set of samples, which served as the primary cohort, was from the Chinese Academy of Medical Sciences (CAMS). For internal validation, we used a computer to randomly select 58 samples from the primary cohort and denoted this set as the internal validation cohort.

For external validation, we first selected samples from the open-access and public TCGA database. Transcriptome sequencing data and follow-up data of 81 samples were downloaded from the TCGA database and denoted as the external validation one cohort**.** A second cohort of 105 ESCC samples for external validation included 38 samples from Anyang Cancer Hospital (ACH), The Fourth Affiliated Hospital of Henan University of Science and Technology, and 67 samples from Henan Key Laboratory of Cancer Epigenetics (HKLCE), The First Affiliated Hospital of Henan University of Science and Technology. This cohort was denoted as external validation two cohort.

### Follow-Up and Classification of Cause of Death

Most of the patients were followed up for 48–72 months. In this study, the statistics were made according to the 4-year survival period. Survivors over 48 months after surgery were counted as living, and survival periods greater than 48 months were calculated as 48 months.

### Study Design

**FIGURE 1:**
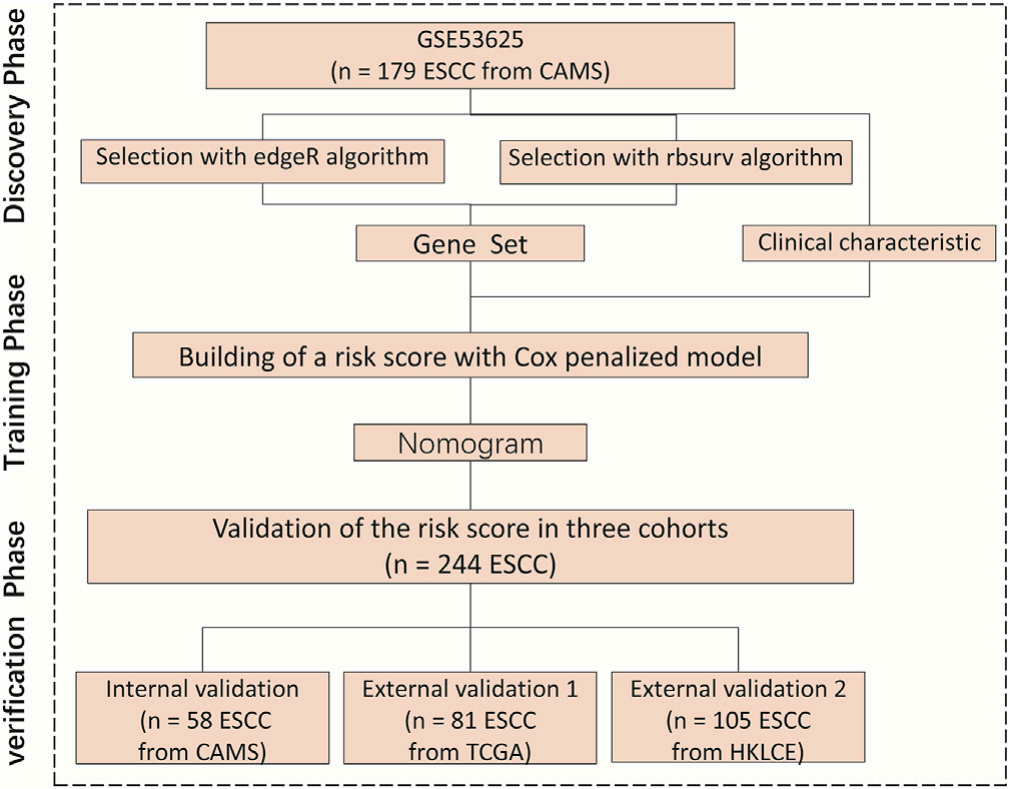
Research flowchart, HKLCE, The First Affiliated Hospital of Henan University of Science and Technology. EdgeR and rbsurv algorithm, School of Information Engineering, Henan University of Science and Technology.

We divided this study into three phases to identify and validate OS-related clinical characteristics and gene sets in ESCC patients. During the discovery phase, we processed the transcriptome sequencing data of the primary cohort to obtain 16,738 genes and then selected important gene sets through two algorithms. During the training phase, a penalized Cox regression model was used to identify the best gene sets (Figure 1). During the verification phase, the gene sets that we chose were validated in the internal validation cohort, external validation one cohort, and external validation two cohort. These three cohorts included patients from multiple medical centers.

Meanwhile, We used qPCR to verify external validation sample 2, in order to obtain results widely used in clinical practice, formalin fixed paraffin embedded samples were used with a minimum tumor cell composition of 80%. Our research program was approved by the ethics committees of the research centers, and 105 samples were reassessed and confirmed by pathologists.

### Dimension Reduction Process

Initially, GSE53625 data were processed with the annotation package to obtain expression spectrum and probe ID. The gene expression value was combined with the annotation file to obtain the complete gene expression value, and the edgeR package was used to find the differential gene. Then, the rbsurv package was used to calculate the dimensionality reduction of the differential gene (max.n.genes = 10, n. iter = 10, n. fold = 3, n. seq = 3, seed = 1,234).

### Construction and Validation of the Nomogram

For the development of the nomogram, we found a number of clinical characteristics that have been shown to be associated with survival as a prognostic characteristic. (Sun et al., 2019) These clinical characteristics (*p* < 0.05) included Age, Sex, Smoking, Drinking, Tumor grade, Tumor location, T stage, N stage, TNM stage, Arrhythmia, Pneumonia, Anastomotic leak, and Adjuvant therapy. For each clinical characteristic, we used a multivariate Cox proportional risk model to evaluate the projected 4-year OS. Nomogram validation was divided into the following three stages. First, internal validation was conducted using the internal validation cohort, and the C-index was estimated by analyzing the area under the curve of the receiver operating characteristic curve. Next, by means of regression analysis, the correction curve was obtained to judge whether the predicted survival probability was consistent with the observed survival probability. (Qiu et al., 2017) The calibration curve adopted Bootstrap resampling (1,000 resampling). Last, external validation was conducted using the external validation cohorts, and Cox regression analysis was performed using the total score of each patient as an independent characteristic. The C-index and calibration curve were obtained by regression analysis.

### Statistical Analyses

Overall survival was defined as the time from the date of initial treatment until the date of death because of ESCC or the date of the last follow-up. The OS curve was estimated by the Kaplan-Meier method and compared with the log-rank test stratified by prognostic factors. The rbsurv packages were used to reduce the dimensions of the data within RStudio Version 1.1.463 software. We built a nomogram in a previous study. On the basis of multivariate Cox analysis results, this nomogram was compiled by R through survival and the RMS software package. (Frank and Harrell, 2014)

## Results

### Clinicopathologic Characteristics of Patients

The research flowchart is shown in Figure 1. The demographics and clinical characteristics of patients with ESCC are presented in Table 1. In the primary cohort, the median follow-up time was 34.7 months (range, 23.4–45.9 months). For the external validation two cohort, of 140 patients with ESCC who received partial esophagectomy during the study period, 105 met the inclusion criteria to enter this study. The median survival time of these 105 patients was 35 months (95% CI, 27.9–42 years). Table 1 lists the demographic and clinicopathological characteristics of patients in the primary cohorts.

**TABLE 1:** Demographics and clinicopathologic characteristics of patients with ESCC.

Characteristics	*N*	Primary cohort (*n* = 179)		
		Hazard ratio	CI95	*p*-value
Age	—	1.62	1.1–2.39	0.015
<60	91	References	—	—
≥60	88	1.6	1.1–2.3	0.02
Sex	—	0.81	0.5–1.31	0.393
Female	33	Reference	—	—
Male	146	0.78	0.49–1.3	0.307
Smoking	—	0.75	0.5–1.11	0.147
No	65	Reference	—	—
Yes	114	0.75	0.37–1.1	0.145
Drinking	—	0.86	0.58–1.27	0.449
No	73	Reference	—	—
Yes	106	1.43	0.79–2.6	0.455
Tumor location	—	1.17	0.86–1.6	0.309
Lower	62	Reference	—	—
Middle	97	1.1	0.74–1.7	0.562
Upper	20	1.7	0.9–3.1	0.101
Tumor grade	—	1.24	1.03–1.48	0.020
Moderately	98	References	—	—
Poorly	49	1.63	1.07–2.5	—
Well	32	0.99	0.57–1.7	—
T stage	—	1.28	0.97–1.69	0.077
T1	12	References	—	—
T2	27	1.1	1.25–2.0	0.863
T3	110	1	0.7–2.3	0.935
T4	30	1.7	0.72–4.0	0.226
N stage	—	1.44	1.18–1.76	<0.001
N0	83	Reference	—	—
N1	62	1.30	0.64–2.6	0.002
N2	22	1.27	0.49–3.3	0.017
N3	12	1.82	0.66–5	0,004
TNM stage	—	2.12	1.47–3.05	<0.001
I	10	Reference	—	—
II	77	1.75	0.55–5.8	—
III	92	3.6	1.14–11.5	—
Arrhythmia	—	1.10	0.71–1.71	0.667
No	126	Reference	—	—
Yes	43	1.1	0.73–0.17	—
Pneumonia	—	1.46	0.74–2.89	0.278
No	164	Reference	—	—
Yes	15	1.4	0.72–2.8	031
Anastomotic leak	—	1.34	0.62–2.9	0.450
No	127	Reference	—	—
Yes	12	1.3	0.63–2.7	0.504
Adjuvant therapy	—	1.38	0.92–2.07	0.115
No	45	Reference	—	—
Unknown	30	2.7	1.4–5.1	0.003
Yes	104	2.3	1.3–3.9	0.003

### Independent Prognostic Factors in the Training Set

The results of the univariable analysis are shown in Table 1. Younger age (<60 vs. ≥60 *p* = 0.015) and TNM stage I (I vs. II vs. III *p* < 0.001) were associated with better prognosis. In addition, age (*p* = 0.015 vs. *p* = 0.037) and TNM stage (*p* < 0.001 vs. *p* = 0.001) were correlated with OS in both the primary cohort (*n* = 179) and the internal validation cohort (*n* = 58).

### Selection of Gene Set

**FIGURE 2:**
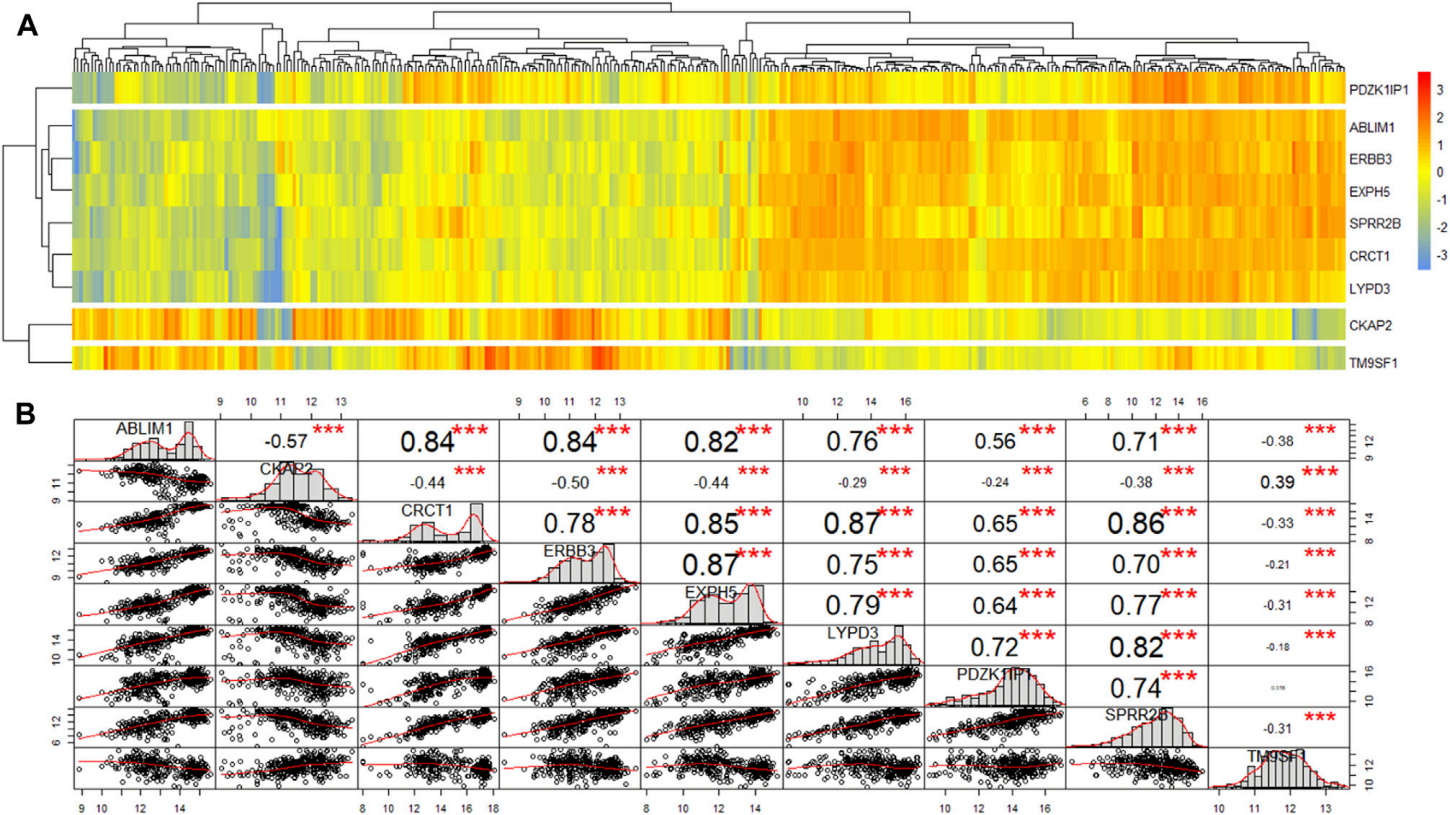
The heatmap and similarity analysis of the expression of nine genes for patients from primary cohort. **(A)** Heatmap demonstrating unsupervised hierarchical clustering of nine genes for patients from primary cohort. **(B)** Similarity analysis of the expression of nine genes for patients from primary cohort.

In total, nine genes were excavated by two algorithms, the edgeR package and rbsurv package. First, heatmap and similarity analysis were performed for these genes. Heatmap demonstrating unsupervised hierarchical clustering of nine genes for patients from primary cohort (Figure 2A). These nine genes were clustered into four groups with obvious differences, among which TM9SF1, CKAP2 and PDZK1IP1 were independently grouped. CKAP2 and TM9SF1 were highly expressed in the left half, while PDZK1IP1 was highly expressed in the right half. Correlation analysis of the nine genes found that they were directly related to each other except for TM9SF1 and PDZK1IP1 (Figure 2B). Three stars in Figure 2B represent *p* values less than 0.001. Figure 2 shows that TM9SF1 and PDZK1IP1 are independent prognostic factors and can be used as marker genes.

Next, we used univariate and multivariate Cox analyses to discriminate the marker genes in the primary cohort (*n* = 179). The results are shown in Table 2. In the univariate cox analysis, all nine genes were correlated with survival, and six genes with *p*-value less than 0.001 were selected for multivariate Cox analysis. Multivariate Cox analysis demonstrated that PDZK1IP1 (high expression vs. low expression, *p* = 0.031) and TM9SF1 (high expression vs. low expression, *p* < 0.001) were independent risk factors for OS (Table 2). Therefore, PDZK1IP1 and TM9SF1 were defined as gene sets directly related to prognosis.

**TABLE 2:** Univariate and multivariate Cox analysis of the expression of nine genes for patients from primary cohort (*n* = 179).

Characteristics	Univariate cox analysis			Multivariate cox analysis		
	Hazard ratio	95% CI	*p*-value	Hazard ratio	95% CI	*p*-value
ABLIM1	0.5	0.34–0.73	**<0.001**	0.75	0.48–1.16	0.191
CKAP2	2.14	1.24–3.71	0.006	—	—	—
CRCT1	0.46	0.30–0.70	**<0.001**	0.80	0.50–1.28	0.349
ERBB3	0.46	0.31–0.68	**<0.001**	0.67	0.43–1.04	0.075
EXPH5	0.4	0.22–0.72	0.002	—	—	—
LYPD3	0.35	0.19–0.64	**<0.001**	0.52	0.26–1.03	0.059
PDZK1IP1	0.3	0.14–0.61	**<0.001**	0.38	0.16–0.91	**0.031**
SPRR2B	0.45	0.27–0.75	0.002	—	—	—
TM9SF1	2.13	1.44–3.14	**<0.001**	2.09	1.38–3.17	**<0.001**

The bold values denoted in Table 2 represent selected marker genes in this study.

**FIGURE 3:**
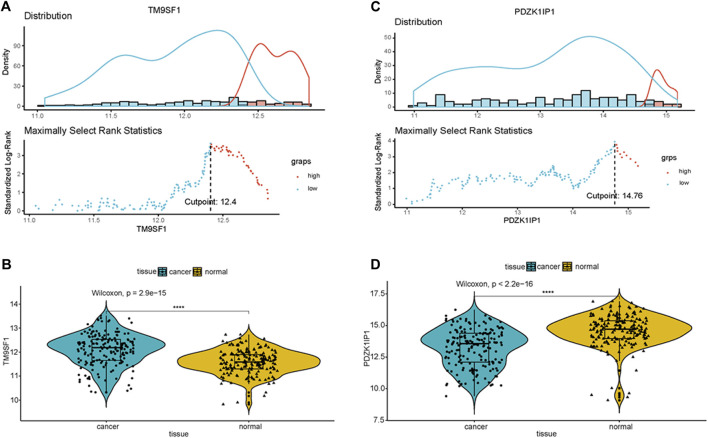
PDZK1IP1 and TM9SF1 were defined as marker genes **(A,C)** Cutpoint of the marker genes was obtained. **(B,D)** Violin plot showed that the expression value of PDZK1IP1 gene was high in normal, TM9SF1 gene has high expression value in cancer.

To classify gene expression values, we determine cutoff values using ggplot2 packages in the primary cohort. The results are listed in Appendix Figure 3A and Figure 3C (TM9SF1 cutpoint = 12.4, PDZK1IP1 cutpoint = 14.78). A violin plot of the marker genes was drawn using primary cohort. The expression value of PDZK1IP1 gene was high in normal, which is significantly different from cancer (Figure 3D, *p* < 0.001). TM9SF1 gene has high expression value in cancer, which is significantly different from normal (Figure 3B, *p* < 0.001).

### Independent Prognostic Factors in the Primary Cohort

Univariate Cox analysis results show that, in the primary cohort, the prognostic factors that predicted poor OS were age ≥60 years, TNM stages II and III, high expression of TM9SF1, and low expression of PDZK1IP1. We performed a multivariate Cox analysis of gene set and clinical clinicopathologic characteristics. The results are listed in Appendix Figure 4. Multivariate Cox analysis demonstrated that age (*p* = 0.031), TNM (*p* = 0.004), PDZK1IP1 (*p* = 0.001), and TM9SF1 (*p* = 0.001) were independent risk factors for OS.

**FIGURE 4:**
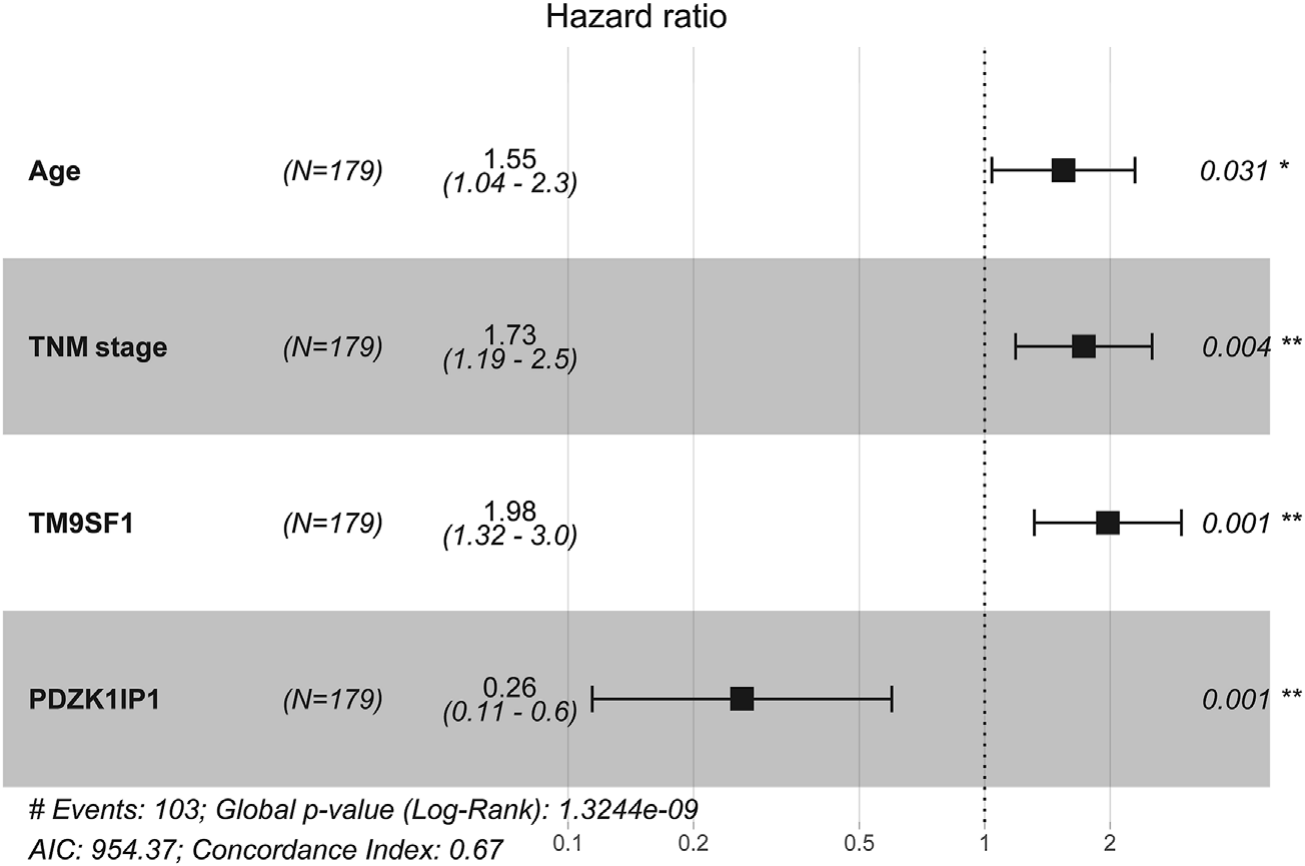
Multivariate cox analysis of the gene set and clinicopathologic characteristics from primary Cohort (*N* = 179).

### Nomogram Development and Validation

**FIGURE 5:**
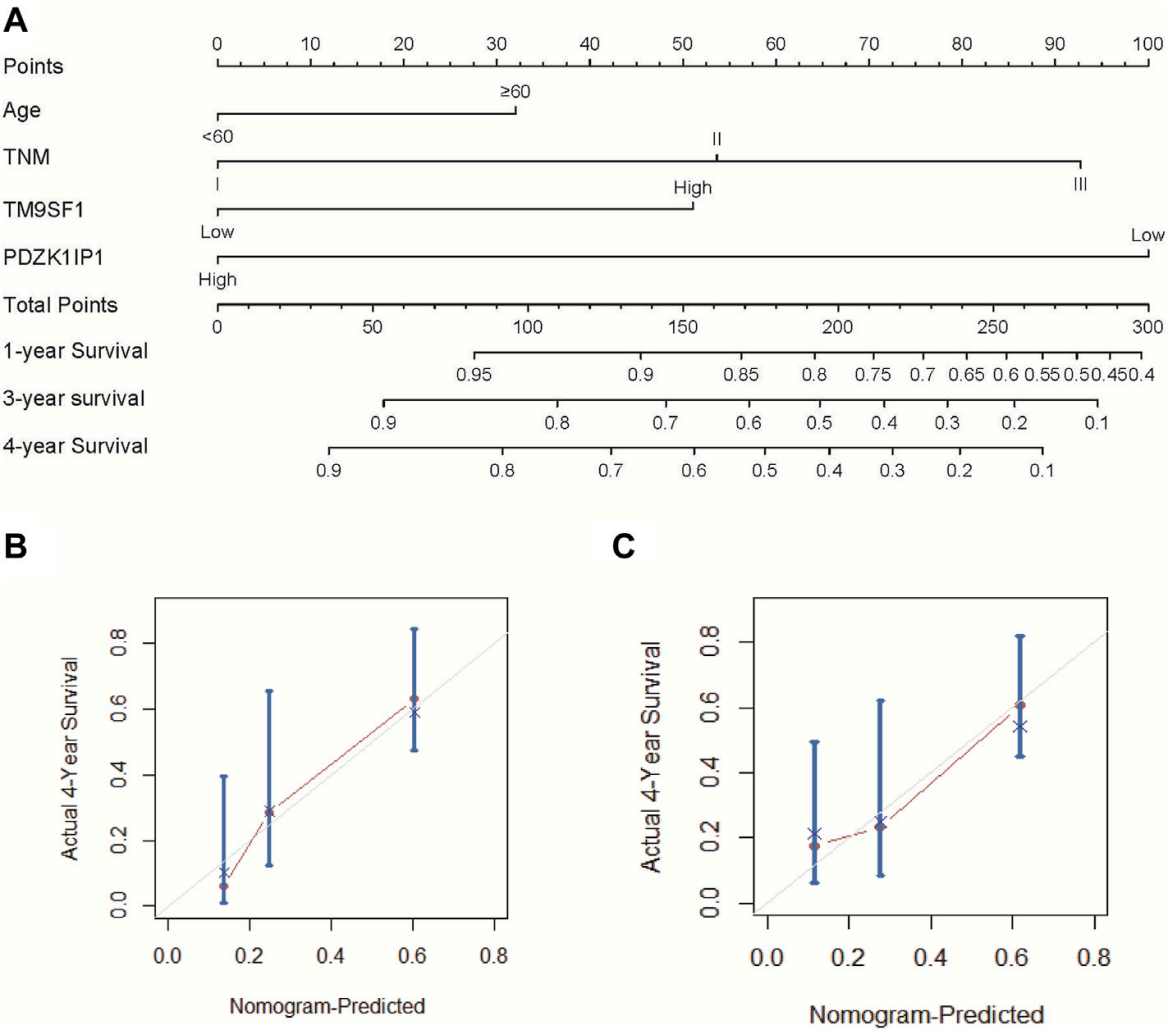
Nomogram and calibration curve of ESCC patients. **(A)** To use the nomogram, each variable has a patient’s assignment on its axis, and a line is drawn upward to determine the number of points for each variable’s value. The sum of these points is located on the total points axis, and then a perpendicular line is then drawn downwards to the survival axis to determine the 1-year, 3-year, and 4-year OS probability. **(B)** The calibration curve for the prediction of 4-year OS based on the internal validation cohort. **(C)** The calibration curve for the prediction of 4-year OS based on the external validation two cohort. In **(B)** and **(C)**, the OS prediction of nomogram probability is plotted on the *x*-axis, and the real OS is plotted on the *y*-axis.

The results of multivariate cox analysis were used to establish a nomogram for the predicted 4-year OS (Figure 5A). As age, TNM, PDZK1IP1, and TM9SF1 were independent risk factors for survival in multivariate Cox analysis, these variables were incorporated into the nomogram. In the internal validation, the predictive accuracy for 4-year OS as indicated by the C-index was 0.75. The 4-year OS probabilistic calibration chart shows that the actual observation results have a high correlation with the prediction results of the nomogram (Figure 5B).

The nomogram was externally verified by the calibration plot in Figure 5C as well as by calculating the bootstrap C statistics of 105 patients in the external validation two cohort. In the external verification stage, the C-index of the 4-year OS nomogram was predicted to be 0.72 (Figure 5C), indicating that the model has a higher discrimination ability. The calibration curve shows that the calibration effect of the nomogram is good; the 4-year OS showed maximum consistency between the actual observation and the nomogram prediction.

### Performance of the Nomogram in Stratifying Risk of Patients

**FIGURE 6:**
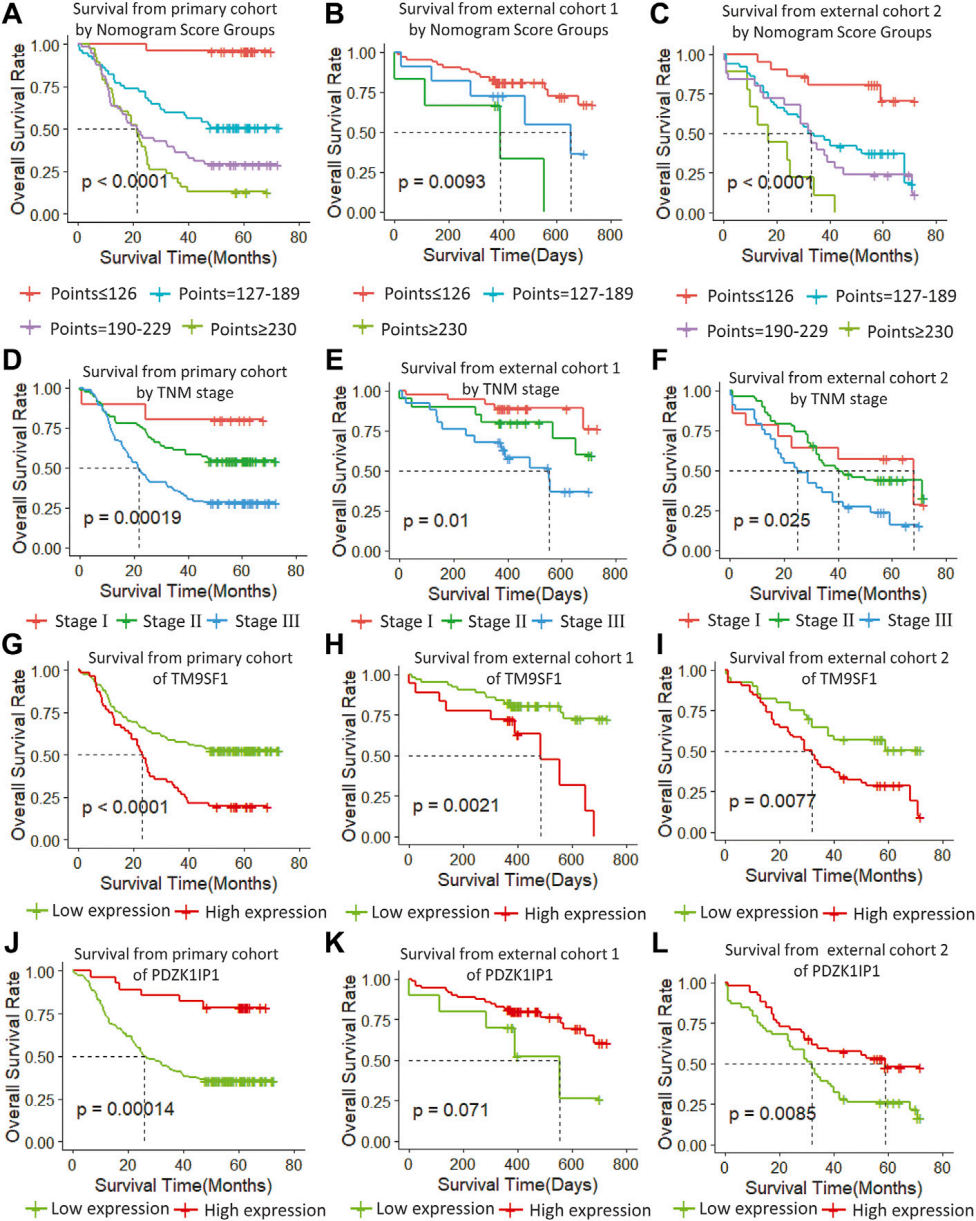
**(A**–**C)** Survival from primary cohort by nomogram score groups, **(D**–**F)** survival from each cohort by TNM stage, **(G**–**I)** survival from each cohort of TM9SF1, and **(J**–**L)** survival from each cohort of PDZK1IP1.

We determined the truncation by dividing the patients in the training cohort into four subgroups based on their total scores (score: 0 to 126, 127 to 189, 190 to 229, and ≥230), where each subgroup corresponds to a different prognosis (Table 3). After applying the cutoff values to sort patients in each cohort, stratification into different risk subgroups resulted in significantly different Kaplan–Meier curves for survival outcomes in each group (Figures 6A–C). The survival curve for subgroups sorted according to TNM stage showed worse performance as shown by the survival rate of stage I being lower than that of stage II (Figure 6F). The grouping result by the nomogram score was observably better than that by TNM stage (*p* < 0.0001 vs. 0.00019, 0.0093 vs. 0.01, <0.0001 vs. 0.025).

**TABLE 3:** Point assignment and prognostic score.

Variable name	Score	Estimated 4-year OS (%)
Age, years	—	—
<60	0	—
≥60	32	—
TNM stage		—
I	0	—
II	54	—
III	93	—
TM9SF1 expression	—	—
High	0	—
Low	51	—
PDZK1IP1 expression		—
High	100	—
Low	0	—
Total prognostic score		
0–126	—	70
127–189	—	44
190–229	—	24
≥230	—	12

**FIGURE 7:**
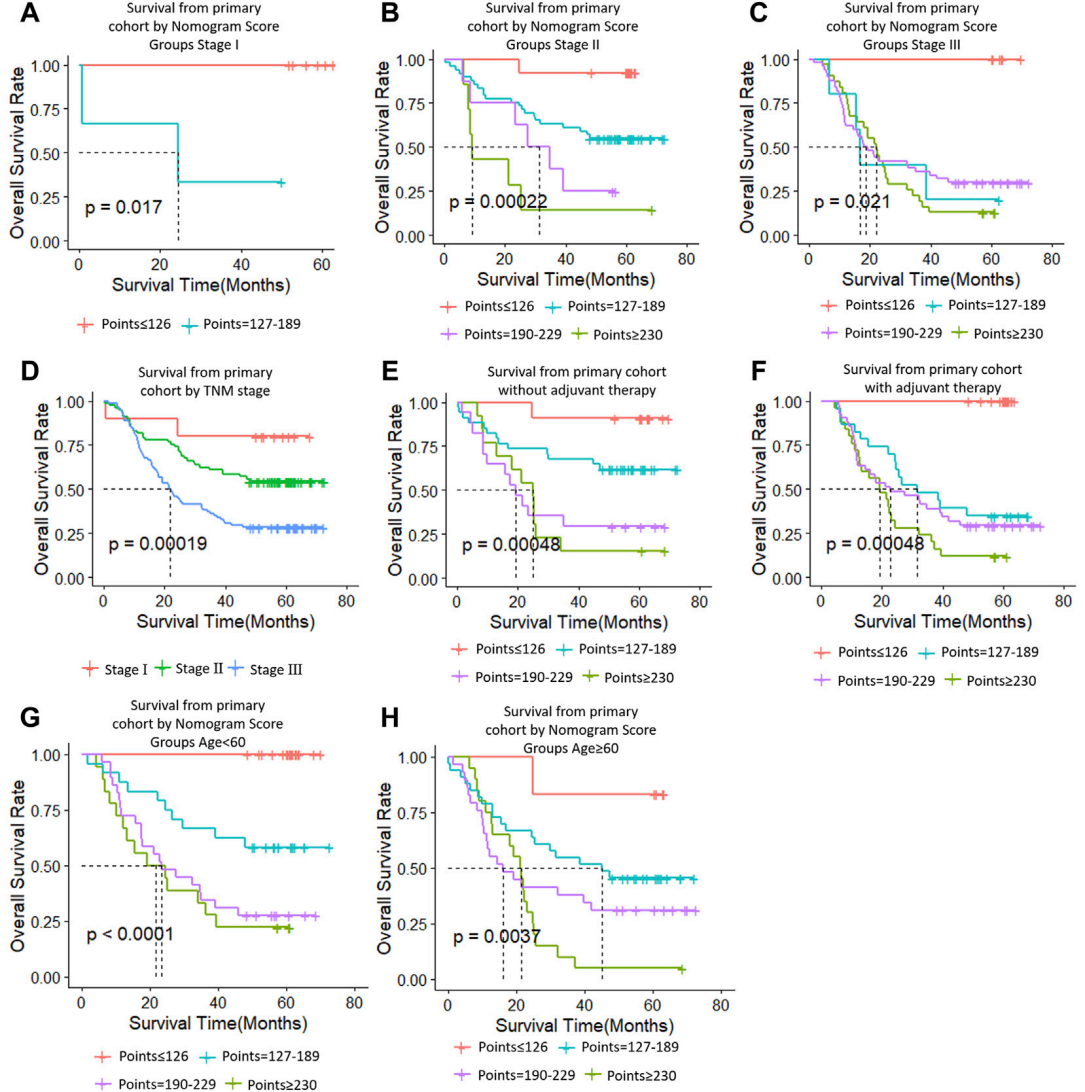
Risk group stratification within each TNM stage (stages, **(A–C)**; **(D)**, all patients), group according to whether receive adjuvant therapy **(E,F)** and group by age **(G,H)**.

At the same time, we performed sub-group analysis of adjuvant therapy and TNM stage in the primary cohort, stratification into different risk subgroups allowed significant distinction between Kaplan-Meier curves for survival outcomes within each TNM stage, age and adjuvant therapy (Figure 7).

### Validating the Marker Genes

In order to verify the stability of the marker genes, we performed validation analysis in the primary cohort and two external validation cohorts. In the primary cohort, we divided the 179 samples into a high TM9SF1 expression group with 56 samples and a low TM9SF1 expression group with 123 samples (*p* < 0.001; Figure 6G). Similar analyses showed that 18 samples with high TM9SF1 expression had poorer prognosis than 63 samples with low TM9SF1 expression in the external validation one cohort (*p* = 0.0021; Figure 6H), and similar results were found in the external validation two cohort (*p* = 0.0077; Figure 6I).

For another marker gene, in the primary cohort, we successfully divided the 179 samples into a high PDZK1IP1 expression group with 28 samples and a low PDZK1IP1 expression group with 151 samples (*p* = 0.0014; Figure 6J). Similar analyses showed that 71 high PDZK1IP1 expression patients had better prognosis than 10 low PDZK1IP1 expression patients in the external validation one cohort (*p* = 0.071; Figure 6K), and 52 high PDZK1IP1 expression samples had better prognosis than 53 low PDZK1IP1 expression samples in the external validation two cohort (*p* = 0.0085; Figure 6L). Together, these results indicate that PDZK1IP1 and TM9SF1 can be defined as marker genes directly related to prognosis.

## Discussion

Due to the significant heterogeneity of ESCC in individual patient survival, the prediction of survival using the TNM staging system is inaccurate. (Sun et al., 2019) It is necessary to develop a new ESCC prognosis grading system. Therefore, we aimed to develop a nomogram model that uses gene expression values to predict long-term survival in patients with operable ESCC.

In this study, we determined that age and TNM stage were independent prognostic factors through univariate and multivariate Cox analyses of clinical characteristics. Age and TNM staging were highly consistent with previous studies on ESCC risk factors. Meanwhile we used edgeR package to screen differential genes and selected nine significant genes by using the rbsurv package in the discovery phase. Then we narrowed the selection down to two marker genes in the training phase. Finally, we verified the marker genes using an internal validation cohort and two external validation cohorts, which included samples from the GEO database and the TCGA database. According to the cutoff value of marker genes, we can divide ESCC cases into two subgroups with significantly different high or low risk of death. ESCC patients with high expression of PDZK1IP1 had worse prognosis than those with low expression, suggesting that PDZK1IP1 was a negative factor for EC mortality (Figures 6G–I). In contrast, TM9SF1 expression levels were correlated with better prognosis, suggesting that TM9SF1 was a positive factor for EC mortality (Figures 6J–L). There have been reports that the TGF-β1,HER-2 and Smad4 are associated with the development of ESCC, and the low quality of HER-2 as a prognostic biomarker in ESCC. (Heidarpour et al., 2020) HER-2 expressed in a variety of tumor tissues including primary breast tumors and tumors from small bowel, esophagus, kidney and mouth. The effect of PDZK1IP1 and TM9SF1 on ESCC is not clear. PDZK1IP1 expressed at significant levels only in a single epithelial cell population, the proximal tubular epithelial cells of the kidney as well as diffusely expressed in various carcinomas originating from kidney, colon, lung and breast. There are reports that PDZK1IP1 interacts with Smad4 and thereby suppresses the TGF-β signaling pathway. (Ikeno et al., 2019) TM9SF1 Plays an essential role in autophagy. There are reports that TM9SF1 as a collaborative EBAG9 interactor, which regulates epithelial-mesenchymal transition (EMT) in cancer cells (Miyazaki et al., 2018).

Therefore, PDZK1IP1 and TM9SF1 are defined as marker genes directly related to prognosis. These two marker genes may have clinical significance for customized follow-up and treatment of ESCC patients. With these two marker genes, low-risk patients can avoid the toxic side effects of adjuvant therapy whereas high-risk ESCC patients can receive more rigorous monitoring and treatment regimens to prevent their condition from worsening (Cao et al., 2016).

The nomogram aimed to estimate 1-year, 3-year, and 4-year OS probabilities based on the multivariate Cox proportional risk model, which includes TNM staging, age, and two mRNA expression values for postoperative measurements of cancer tissue. In the validation phase, we demonstrated that the nomogram was an excellent model for predicting 1-year, 3-year, and 4-year OS for ESCC patients, and we demonstrated that the accuracy was better than TNM staging. The predicative accuracy of our nomogram model in primary cohort is the best one among PDZK1ID1, TM9SF1, Age, TNM as single predictors with C-index of 0.75, 0.701, 0.659 and 0.65 (Table 4). All of the aforementioned predictors have greater C-index compared with age with a C-index of 0.532. In external cohort one and cohort two, similar results were obtained. In addition, the two clinicopathologic characteristic and the mRNA expression values of two genes incorporated into nomogram should be recorded by every clinician for ESCC patients to increase their clinical effectiveness.

**TABLE 4:** Prognostic ability and accuracy of ESCC.

Factor	Primary cohort		External cohort 1		External cohort 2	
	*p*-value	C-index	*p*-value	C-index	*p*-value	C-index
Nomogram	<0.0001	0.75	0.0093	0.695	<0.0001	0.72
PDZK1ID1	0.00014	0.701	0.071	0.661	0.0086	0.639
TM9SF1	<0.0001	0.659	0.0021	0.52	0.0077	0.656
Age	0.015	0.532	0.4	0.376	0.061	0.596
TNM	0.00019	0.65	0.01	0.6	0.025	0.630

To further evaluate the predictive capacity and accuracy Nomogram. The higher C-index score represent the better prognostic performance of the system.

In this study, the proposed nomogram was verified to avoid overfitting of the model and to determine the generalization of the model. (Iasonos et al., 2008) The predicted value of the calibration diagram was highly consistent with the actual value, which proved the accuracy and repeatability of the nomogram. Furthermore, the model was validated using the TCGA database (external validation one cohort), which included patients from Asia, North America, South America, and Europe, making it possible to use and promote the model globally, regardless of race, regional lifestyle, and economic factors. In the verification phase, the C-index of the model was obviously better than the TNM staging system. Using the model, the OS rate prediction ability was slightly worse in the external validation one cohort (Figure 6B) compared to the primary cohort (Figure 6A) and the external validation two cohort (Figure 6C). Subsequently, applying threshold values to divide each cohort into four different risk subgroups resulted in significant differences in Kaplan–Meier curves for survival results in every group. The discrimination ability of the primary cohort (C-index, 0.75 for nomogram vs. 0.68 for TNM staging system; 0.07 difference) and external validation two cohort (0.72 for nomogram vs. 0.64 for TNM staging system; 0.08 difference) were similar. When using the TNM staging system, the survival curve of patients with stage II did not reach the significance level (Figures 6D–F). Additionally, there were some intersections among the OS rate lines of different nomogram score groups (Figure 6C) and TNM stage groups (Figure 6F). We think that sample size is the most important reason for these indistinctions between different nomogram score groups and TNM stage groups.

In recent years, researchers have used nomograms to predict ESCC, and this study has several advantages over previous studies. First, to avoid specificity, we confirmed the gene markers in samples from multiple medical centers. Second, the majority of previous studies did not consider mRNA in ESCC. Marker genes play an important role in the development of ESCC, and their differential expressions are associated with the development of ESCC. Finally, we adopted a combinatorial strategy in our study, which is different from previous studies that used only one algorithm to select markers. The purpose of the combinatorial algorithm is to reduce the possibility of missing or ignoring important marker genes.

The limits of this study includes the following points. First, we used limited sample sizes in the training and test cohorts that results in some discrepancy. Second, all ESCC patients in primary cohort are Chinese origin contrasting with ESCC patients from TCGA in external cohort one across world. Therefore, the predictative power of our nomogram model in ESCC patients from TCGA is less efficient compared with primary and external cohort two. We speculate that the different predicative capability is ascribed to regional difference of ESCC cases. Third, our nomogram predicts overall survival well for subgroup patients with or without adjuvant therapy, indicating that it is not appropriate for decision-making on adjuvant therapy (Supplementary Figure S1). The underlying causes are not clear at present and warrants further study to investigate.

In summary, the nomogram presented in this study can accurately and impersonally predict the prognosis of ESCC patients after partial resection of the esophagus. More research is required to determine whether it can be applied to other patient populations. Moreover, we found two marker genes directly related to the prognosis of ESCC, which will provide a basis for future research.

## Data Availability

The original contributions presented in the study are included in the article/Supplementary Material, further inquiries can be directed to the corresponding authors.
