# Supplementary figures and images for: A Prognostic Model Based on mRNA Expression Analysis of Esophageal Squamous Cell Carcinoma
Source: Front Bioeng Biotechnol. 2022 Mar 1;10:823619. doi: 10.3389/fbioe.2022.823619 (PMC8921680; doi:10.3389/fbioe.2022.823619)

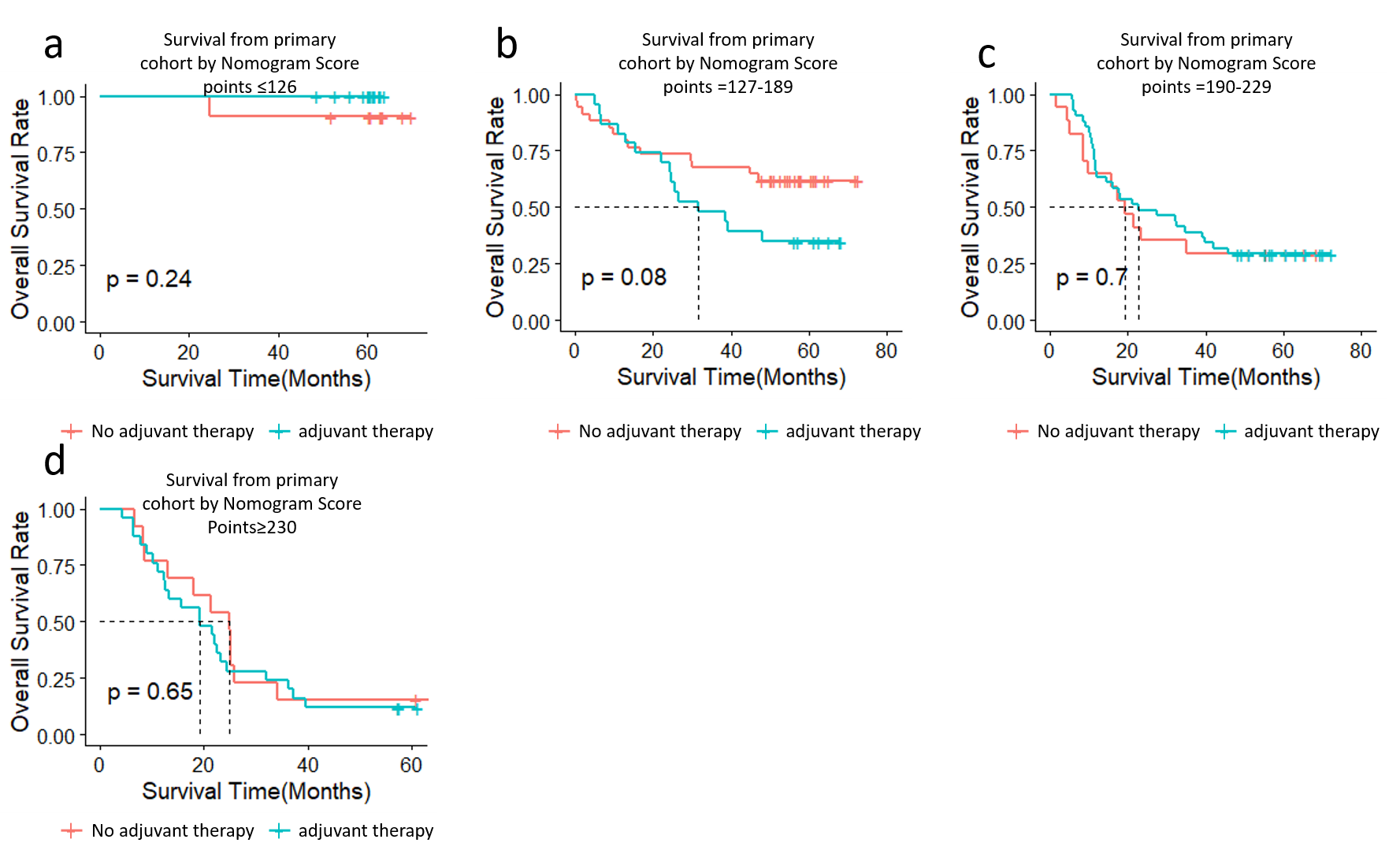

Supplement: Supplementary file 1 [file Image1.tif]
